# Challenges in Promoting Joint Use Agreements: Experiences From Community Transformation Grant Awardees in North Carolina, Illinois, and Wisconsin, 2011–2014

**DOI:** 10.5888/pcd12.140457

**Published:** 2015-04-16

**Authors:** Anna Stein, William Baldyga, Amy Hilgendorf, Jennifer Gilchrist Walker, Danielle Hewson, Lori Rhew, Amber Uskali

**Affiliations:** Author Affiliations: William Baldyga, Amber Uskali, University of Illinois at Chicago, Chicago, Illinois; Amy Hilgendorf, Jennifer Gilchrist Walker, University of Wisconsin-Madison, Madison, Wisconsin; Danielle Hewson, Lori Rhew, North Carolina Division of Public Health, Raleigh, North Carolina.

## Abstract

Community Transformation Grant awardees in North Carolina, Illinois, and Wisconsin promoted joint use agreements (formal agreements between 2 parties for the shared use of land or facilities) as a strategy to increase access to physical activity in their states. However, awardees experienced significant barriers to establishing joint use agreements, including 1) confusion about terminology and an aversion to complex legal contracts, 2) lack of applicability to single organizations with open use policies, and 3) questionable value in nonurban areas where open lands for physical activity are often available and where the need is instead for physical activity programs and infrastructure. Furthermore, promotion of formal agreements may unintentionally reduce access by raising concerns regarding legal risks and costs associated with existing shared use of land. Thus, joint use agreements have practical limitations that should be considered when selecting among strategies to promote physical activity participation.

## Introduction

Chronic diseases are among the leading causes of death and disability in the United States, yet it is widely recognized that they are largely preventable (1,2). Because of its established health risks and substantial increases in prevalence, obesity has become a major national and global health challenge (3). Reducing obesity requires successful implementation of evidence-based practices demonstrated to improve health outcomes, including interventions to increase physical activity (4). The Community Preventive Services Task Force recommends environmental and policy changes to increase physical activity, including creating or enhancing access to places for people to be active (5).

In 2011, the Centers for Disease Control and Prevention (CDC) awarded grants to 61 state and local government agencies, tribes, territories, and nonprofit organizations in 36 states as part of its Community Transformation Grant (CTG) program (6). Through a regional or county-level approach, CTG awardees implemented action plans over a 3-year period in the following strategic areas: active living and healthy eating, tobacco-free living, clinical and community preventive services, healthy and safe physical environment, and social and emotional wellness (6).

One of the strategies chosen by CTG grantees working on active living strategies was to increase the number of joint use agreements (JUAs) to promote community access to places for physical activity. JUAs are formal agreements between 2 separate entities — often a school and a city or county — setting forth the terms and conditions for shared use of property or facilities (7). JUAs have been identified as a promising strategy for increasing access to existing recreational facilities, especially for people who do not have access to safe places to be active (8–11).

Initial JUA research has focused on the identification of barriers to their implementation, particularly concerns about liability (12–14). Investigation into the impact of JUAs on physical activity participation is limited. Lafleur and colleagues, evaluating Los Angeles school districts with limited access to open space, demonstrated that JUAs were conducive to increased physical activity, particularly when programs such as organized sports were offered (15). No research has yet been conducted on the effectiveness of the JUA strategy in nonurban areas.

In 2013, representatives from several CTG awardee states requested technical assistance from CDC in the form of a list of contacts in other states who were working on the JUA strategy. These representatives then convened a series of conference calls with multiple state participants, leading to recognition that they faced similar barriers in pursuing the JUA strategy. CTG staff from 3 of these states — North Carolina, Illinois, and Wisconsin — continued these discussions, leading to the development of this article. In these 3 states, the JUA strategy was pursued at the local level by CTG-funded staff in county health departments or community-based organizations (Table). These staff initiated and facilitated discussions about JUAs with community partners, although the implementation of JUAs was largely the responsibility of representatives of those institutions. We reviewed written performance reports and had conversations with local CTG awardees in the 3 states in preparation for writing this article. We share our respective states’ CTG experiences with the JUA strategy, concluding with a synthesis of lessons learned to help inform practitioners and researchers.

**Table:** Community Transformation Grant (CTG) Awardees in North Carolina, Illinois, and Wisconsin, 2011–2014

CTG State Awardee	Local Awardees	MSA Designation^a^ of Counties Covered by Local Awardees	Intended Targets of Local Awardees’ JUA Strategy
North Carolina Division of Public Health	Noncompetitively awarded to LHDs covering 98 of 100 counties (2 most populous counties excluded); administratively divided into 10 regions with 1 LHD serving as lead for each region	44 MSA counties and 54 non-MSA counties	Various local institutions and organizations (eg, school districts, individual schools, community colleges, faith-based organizations, parks and recreation departments)
Illinois Department of Public Health	Competitively awarded to 6 LHDs and LHD coalitions, representing 13 counties	3 MSA counties and 10 non-MSA counties	Various local institutions and organizations (eg, individual schools, community colleges, faith-based organizations, parks and recreation departments)
Wisconsin Clearinghouse for Prevention Resources	Competitively awarded to health coalitions in 14 counties; composition of coalitions included LHDs, nonprofit organizations, school districts, university extension offices	8 MSA counties and 6 non-MSA counties	School settings, including school districts and individual schools

Abbreviations: JUA, joint use agreement; LHD, local health department; MSA, metropolitan statistical area.

a Office of Management and Budget (23).

### North Carolina

The North Carolina Division of Public Health conducted its CTG project by providing awards to local regions encompassing 98 of the state’s 100 counties. As North Carolina’s CTG awardees worked to increase the number of JUAs in the state, they encountered numerous barriers, including confusion over terminology, a lack of practical shared use models, and concerns about formalizing informal arrangements.

An initial barrier was confusion over the scope of the term “JUA.” Although JUAs and “open use policies” are both forms of “shared use,” they are distinguishable in that the former are contracts between at least 2 parties to share space, and the latter arise when one organization — typically a school or faith-based organization — provides free access to the public to its facilities or grounds for physical activity (16). When CTG began, neither state nor local staff fully appreciated this difference in types of shared use, but they came to understand that many of their successes actually involved the implementation of open use policies, not JUAs. This was a critical realization, since CTG grantees had been charged by CDC with promoting JUAs. State CTG staff consequently sought and received approval from the CDC project officer to broaden the strategy and its measures to include one-party open use polices, in addition to multiple-party JUAs.

Another barrier cited by local awardees was the dearth of model contracts and policies for shared use. The only shared use models North Carolina found in the literature when CTG began in 2011 were 4 JUAs written by ChangeLab Solutions (7), all of which were contracts between a school system and a city or county government. Partners in the field complained to awardees that these model contracts were too long and complicated to adapt for local use. They also desired access to examples of real-world JUAs that had been implemented successfully. Furthermore, awardees wanted models to use when approaching faith-based organizations and other nonprofits, including one-party open use policies to assist organizations opening up space on their own. As a result of North Carolina’s request for technical assistance through CTG, in 2014 ChangeLab Solutions produced a fact sheet to educate faith-based organizations about their options for sharing use of their facilities with community members beyond their congregations. The fact sheet included a model open use policy (17).

Finally, some local awardees encountered resistance to formalizing existing shared use arrangements. Many North Carolina schools have long allowed the public to use their grounds for physical activity through unofficial open use policies, but some administrators were reluctant to put a formal open use policy into place because of concern that officially condoning the public’s use of the property might increase the school’s liability risk. In these cases, it was difficult for awardees to know if they should continue working toward a formalized policy at the risk of having the school board decide to terminate all public use of the property, thereby creating the unintended effect of actually reducing access to publicly available recreational spaces. Some local awardees worked with schools to develop signage welcoming the public to play on their playgrounds as a way to formalize use even without accompanying formal policies opening their grounds. To maximize public access to school property for physical activity, legislation may be needed that officially 1) recognizes schools’ authority to open grounds to the public through open use policies and 2) clarifies that schools have governmental immunity for injuries occurring during these uses (18).

### Illinois

The Illinois Department of Public Health awarded CTG funds for JUA development to 6 local health departments, representing 13 primarily rural and suburban counties. Local awardees received technical assistance and consultation on JUA development from the Active Transportation Alliance, a nonprofit organization promoting active living and safe transportation. These awardees reported common issues regarding the applicability and acceptance of JUAs in their communities.

Local awardees assessed physical activity opportunities in their communities and reported the widespread availability of unfenced lands (including school grounds) open to the public for physical activity, pointing to a questionable need for JUAs to provide public access. When discussing the possibility of implementing formal policies to replace existing informal arrangements, as in North Carolina, some local administrators and officials were unwilling to engage in negotiations that might raise potentially contentious issues such as liability. School, park, and other facility administrators rejected the argument that formalizing arrangements would better ensure universal availability and sustainability and saw benefit in maintaining the flexibility of existing informal arrangements.

Several local awardees reported greater interest and enthusiasm on the part of community representatives for strategies that promote physical activity program opportunities. These awardees identified local interest in developing communication about the availability of existing community resources; creating new physical activity infrastructure, such as walking and biking trails; and organizing community walking and biking groups and events. Local awardees also reported unrealized opportunities to offer no- or low-cost programs, for example, by using local exercise instructors to offer classes such as aerobics, yoga, and Tai Chi in underused spaces at community colleges, technical schools, and vacant stores. However, because such pursuits did not qualify as JUAs under guidance issued by CDC, local awardees did not explore them further. Although JUAs were often not viable options for the Illinois CTG awardees, participation in the CTG process did foster conversation about promoting community physical activity among local stakeholders.

### Wisconsin

As part of its CTG project, the Wisconsin Clearinghouse for Prevention Resources funded 14 county-based coalitions to create more opportunities for physical activity in local communities. Grantees worked to establish and sustain JUAs between school districts or individual schools and community partners and encourage development of associated programming. Wisconsin’s CTG work was heavily influenced by the passage of the 2011 Wisconsin Act 162, or the “Open Gym Act,” which expanded schools’ liability protections for after-hours community use of indoor spaces (protection for use of outdoor spaces was already in place) (19). However, several awardees’ reports suggested that aspects of the Open Gym Act may have hindered their abilities to implement JUAs to affect physical activity meaningfully.

Two particular limitations of the Open Gym Act challenged local implementation. First, the law does not provide automatic liability protection, but instead requires schools to include specific information in JUAs, such as a description of the activities to be held on school grounds, to receive liability protection (19). As a result, local awardees often worked with schools to revise existing agreements to add compliant language. After the time and effort spent in these revisions, awardees sometimes found little interest among school administrators in developing new JUAs or programming — efforts that would actually expand physical activity opportunities for community members. Second, because the law did not eliminate liability but rather shifted the burden to community partners, awardees had difficulty finding partners willing to take on that liability, especially in disadvantaged rural or low-income communities.

Ultimately, the Open Gym Act’s liability protections failed to sufficiently motivate many school or community partners to substantially change the use of public spaces. Schools argued that their spaces were already used to capacity or cited cost concerns of extended hours. Schools that did update their policies to comply with the law often continued using spaces as before. New programs, when developed, more often expanded physical activity opportunities for school youths in after-school programs rather than create new opportunities for other community members, especially low-income adults or other underserved groups.

However, by initially discussing JUAs with schools and community partners, some awardees were able to pursue other initiatives that promoted physical activity. For instance, some local awardees helped expand public access to physical activity opportunities in community centers and faith-based or other organizations. Some awardees used Participatory Photo Mapping (PPM) (20) to learn of community members’ interests and priorities for supporting physical activity. PPM illuminated community interest in solutions such as safer routes to school (eg, improved sidewalks and bike lanes, traffic calming measures), enhanced park facilities, and community events (eg, bicycle rides, snowshoeing). As in North Carolina and Illinois, these experiences suggest that JUAs may be too narrow a strategy for promoting physical activity in many communities.

## Discussion

Several themes emerged from the experiences of North Carolina, Illinois, and Wisconsin in implementing the CTG strategy to increase the number of JUAs. First, awardees learned that JUAs are imperfect tools for public health practitioners to increase access to places for physical activity. JUAs are by their nature detailed documents that spell out the parties’ future obligations, and their length and complexity are often intimidating to community partners, particularly when legal support is not readily available. Also JUAs do not resolve the costs of providing access to the public — namely, the costs of liability insurance, additional staffing, and facility upkeep. They merely pass the costs on to another party, and if the party seeking to use facilities lacks sufficient resources to pay for the added costs, a JUA is not feasible. This financial barrier could have particular negative implications for low-resource communities. Additionally, because JUAs are contracts between at least 2 parties, JUAs are not relevant if one organization is willing to open space to the public on its own — an open use policy is instead appropriate. In sum, JUAs are fundamentally complicated, do not eliminate cost concerns, and are useful only when 2 parties are involved.

Second, it is possible that pursuing formal agreements or policies can result in the unintended consequence of reducing access to spaces for physical activity. Some community partners expressed reluctance to commit in writing to do something they had already been doing in practice. Discussing issues of liability and maintenance with facility managers raised their level of concern for the appropriateness of existing open use arrangements. Keeping in mind the goal of maintaining and increasing access, it may be wise not to insist on formal arrangements when informal ones provide sufficient access.

Third, a singular emphasis on implementing JUAs may ignore other, more locally effective strategies for increasing physical activity. In North Carolina, Illinois, and Wisconsin, CTG awardees found that many communities do not lack open space. Particularly in nonurban areas, school playgrounds are often unfenced and community partners such as faith-based organizations allow community access to spaces for physical activity. Awardees expressed more urgent needs in these communities for infrastructure improvements, such as playground equipment, walking trails, bike paths, and sidewalks, and for improved promotion of existing resources. Moreover, awardees identified the need to link program opportunities with already accessible community facilities to effectively increase physical activity participation. This recommendation is consistent with the finding from Lafleur and colleagues that community members’ use of sites was 16 times higher in joint use schools that had physical activity programs than in schools without such programs (15) and with other research showing that physical activity programs increase both use of space and activity levels (21,22). Whether agreements or policies opening access to space can increase physical activity participation without the addition of physical activity programs remains an open question.

Flexible approaches to increasing physical activity in diverse geographic settings are needed. Strategies should ideally be guided by the needs of the community, with a tailored approach to promoting shared use and maximizing use of spaces for physical activity. Further research is needed to measure the actual impact of JUAs and other forms of shared use on physical activity participation by community members, including whether access to facilities alone is sufficient to increase physical activity without additional programs and on the relative effectiveness of formal versus informal agreements or policies to share space.
